# Supplementary material for: Correspondence between cerebral glucose metabolism and BOLD reveals relative power and cost in human brain
Source: Nat Commun. 2019 Feb 11;10:690. doi: 10.1038/s41467-019-08546-x (PMC6370887; doi:10.1038/s41467-019-08546-x)
Supplement: Supplementary file 1 — Supplementary Information [file 41467_2019_8546_MOESM1_ESM.docx]

**Supplementary Information**

**Correspondence between cerebral glucose metabolism and BOLD reveals relative power and cost in human brain**

**Shokri-Kojori et al.**

**Supplementary Results**

**Generalizability** **of rPWR and rCST**

We tested whether rPWR and rCST are generalizable to alternative measures of neuronal activity (i.e., fALFF) and metabolic supply (i.e., CBF) in cohort-1. We used cerebral blood flow (CBF) as an alternative proxy of cerebral metabolic supply[^1^](#_ENREF_1). Consistent with prior reports (Supplementary Fig. 5a), CMRglc and CBF (indexed by perfusion-weighted imaging: PWI) (see Methods) showed a good correspondence across brain networks in our study (Supplementary Fig. 5b). lFCD and fALFF were also highly correlated across networks (Supplementary Fig. 5c), but neither were significantly correlated with CMRglc or with CBF (PWI) across networks (Supplementary Fig. 5d–f). There was an excellent agreement between rPWR of different networks when estimated using lFCD-CMRglc and when estimated using fALFF-CBF (PWI) (intraclass correlation: ICC(3, 1) = 0.8, Supplementary Fig. 6a). Only using CBF (PWI) instead of CMRglc also showed strong agreement in rPWR estimates (ICC(3, 1) = 0.85, Supplementary Fig. 6c). The lFCD threshold had minimal impact on rPWR estimates (ICC(3, 1) = 0.99, Supplementary Fig. 6d). Similarly, there was an excellent agreement between rCST of different networks when estimated using lFCD-CMRglc and when estimated using fALFF-CBF (PWI) (ICC(3, 1) = 0.87, Supplementary Fig. 7a). Changing lFCD to fALFF had minimal impact on rCST estimates (ICC(3, 1) = 0.99, Supplementary Fig. 7b), so did using CBF (PWI) instead of CMRglc (ICC(3, 1) = 0.85, Supplementary Fig. 7c). The lFCD threshold had minimal impact on rCST estimates, as well (ICC(3, 1) = 0.99, Supplementary Fig. 7d).

**Brain morphometry and temporal signal to noise ratio**

In 34 bilateral ROIs in cohort-1 (see Methods), we studied how lFCD, CMRglc, rPWR, and rCST (Supplementary Table 8, also see Supplementary Fig. 1) were associated with brain morphometry (i.e., cortical thickness and cortical distance) and temporal signal to noise ratio (tSNR) of fMRI and FDG-PET, within and between subjects. Supplementary Tables 9–12 show these correlations across subjects for each ROI, while also reporting the level of significance for the trend of correlations across ROIs. Across ROIs, there was a positive trend of correlations between cortical thickness and lFCD (*p* < 0.0001) and between cortical thickness and CMRglc (*p* < 0.0001), but not with rPWR nor with rCST (Supplementary Table 9). rPWR in superior frontal ROI was associated with cortical thickness (*p* < 0.05, Bonferroni). Cortical distance (average geometrical distance between an ROI to other ROIs, see Methods) showed a significant trend of negative correlations across ROIs with CMRglc (Supplementary Table 10). Cortical distance was significantly associated with rCST in entorhinal, lateral orbitofrontal, and inferior temporal ROIs and with rPWR in pars triangularis (*p* < 0.05, Bonferroni).

For fMRI, tSNR (see Methods) was negatively associated with lFCD (*p* = 0.005) and positively with CMRglc (*p* = 0.0004) across ROIs (Supplementary Table 11), but not with rPWR nor with rCST. For FDG-PET, tSNR (see Methods) was negatively associated with lFCD (*p* = 0.02) and positively with CMRglc (*p* < 0.0001) (Supplementary Table 12). FDG-PET tSNR was significantly associated with rPWR (*p* = 0.02) and rCST (*p* = 0.03), but to a weaker extent than the associations between FDG-PET tSNR and CMRglc (*p* < 0.0001, Supplementary Table 12). While temporal pole and entorhinal ROIs both had the lowest tSNR in FDG-PET and fMRI, their rPWR and rCST were not associated with tSNRs (Supplementary Tables 11, 12). In fact, rPWR and rCST in none of the ROIs were associated with tSNRs after correction for multiple comparisons. We also used fMRI tSNR and FDG-PET tSNR maps to compute SNR-based rPWR and rCST maps and performed cortical segmentation with *k*-means clustering (*k* = 4, Supplementary Fig. 8) and found that the clusters differed from those based on lFCD-CMRglc.

**Supplementary Methods**

**MRI and PET data preprocessing**

For cohort-1, MRI data were processed using the minimal preprocessing pipeline of the Human Connectome Project[^2^](#_ENREF_2). FreeSurfer v5.3 (Martinos Center for Biomedical Imaging, Charlestown, Massachusetts, USA; https://surfer.nmr.mgh.harvard.edu/) was used for anatomical segmentation, anatomical parcellation[^3^](#_ENREF_3), and measuring surface-based cortical thickness[^4^](#_ENREF_4). In addition, each fMRI session data underwent gradient distortion correction, rigid body realignment, field map processing, spatial normalization to the stereotactic space of the Montreal Neurological Institute (MNI) with 2-mm isotropic resolution, and brain masking using routines from University of Oxford's Center for Functional Magnetic Resonance Imaging of the Brain Software Library (FSL) release 5.0 (<http://www.fmrib.ox.ac.uk/fsl>). CMRglc is theoretically a composite measure of apparent influx rate (*K*_1_), efflux rate (*k*_2_), phosphorylation rate (*k*_3_), and dephosphorylation rate (*k*_4_), glucose concentration in plasma (*C*_p_) and the lumped constant (*LC*)[^5^](#_ENREF_5)^,^ [^6^](#_ENREF_6). Voxel-level CMRglc (μmol×min^-1^×100 g^-1^) was computed in PMOD v3.4 (PMOD Technologies, Zurich, Switzerland), based on an autoradiographic solution for the 2-tissue compartment model[^6^](#_ENREF_6) up to the mid-time of the summary image (55 min). Gray matter parameters were *K*_1_ = 0.102 min^-1^, *k*_2_ = 0.130 min^-1^, *k*_3_ = 0.062 min^-1^, *k*_4_ = 0.0068 min^-1^, and *LC* = 0.52. CMRglc and CBF (PWI) maps were aligned to individual subject’s anatomical space and were normalized to MNI space using FSL parameters.

For cohort-2, fMRI time series were realigned and normalized to the MNI space with 3-mm isotropic voxels in SPM8 (Wellcome Trust Centre for Neuroimaging, London)[^7^](#_ENREF_7). There were no significant main effects of Group or Alcohol, or a significant interaction effect on motion estimates (*p* > 0.05)[^8^](#_ENREF_8). CMRglc images were computed using the Sokoloff’s model[^9^](#_ENREF_9)^,^ [^10^](#_ENREF_10). CMRglc maps were normalized to the MNI space in SPM8.

**Supplementary Discussion**

FDG-PET is a reliable method for measuring CMRglc in the human brain. In comparison, there are many metrics available to assess voxel-level functional activity during resting-state fMRI. While some metrics focus on regional synchrony in slow (< 0.1 Hz) BOLD fluctuations as a marker of neuronal activity (e.g., lFCD[^12^](#_ENREF_12) or regional homogeneity measures[^13^](#_ENREF_13)), others focus on the amplitude of low frequency fluctuations (e.g., ALFF or fALFF) during resting state[^14^](#_ENREF_14)^,^ [^15^](#_ENREF_15). Synchrony-based and amplitude-based measures appear to capture overlapping and nonoverlapping aspects of brain activity. For example, they have shown good correspondence at rest[^16^](#_ENREF_16) and between different subject groups[^17^](#_ENREF_17). Our results indicated an excellent agreement between lFCD-based and fALFF-based rPWR estimates and between lFCD-based and fALFF-based rCST estimates, across major brain networks (Supplementary Figs. 6c and 7c). However, there are indications that synchrony-based measures may be more sensitive to changes in functional activity (e.g., eyes open versus closed) than amplitude-based measures[^18^](#_ENREF_18). These observations are consistent with the relatively high spatial spread of neuronal activity with respect to the stimulation locus[^19^](#_ENREF_19)^,^ [^20^](#_ENREF_20) that could be captured with synchrony-based measures of brain activity.

**
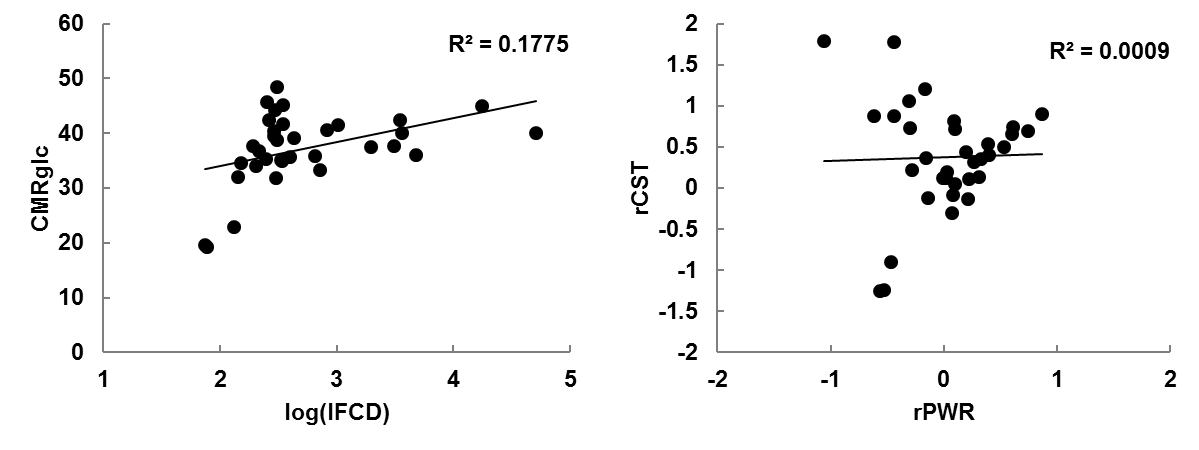
**

**b**

**a**

**Supplementary Figure 1.** Correlation between lFCD and CMRglc across 34 bilateral cortical parcellations (see Methods) (*r*(32) = 0.42, *p* = 0.01) (**a**), and between rPWR and rCST (*r*(32) = 0.03, *p* = 0.87) (**b**) in cohort-1. Across the 34 bilateral ROIs the coefficient of variation of lFCD was 23% and coefficient of variation of CMRglc was 18%.

**
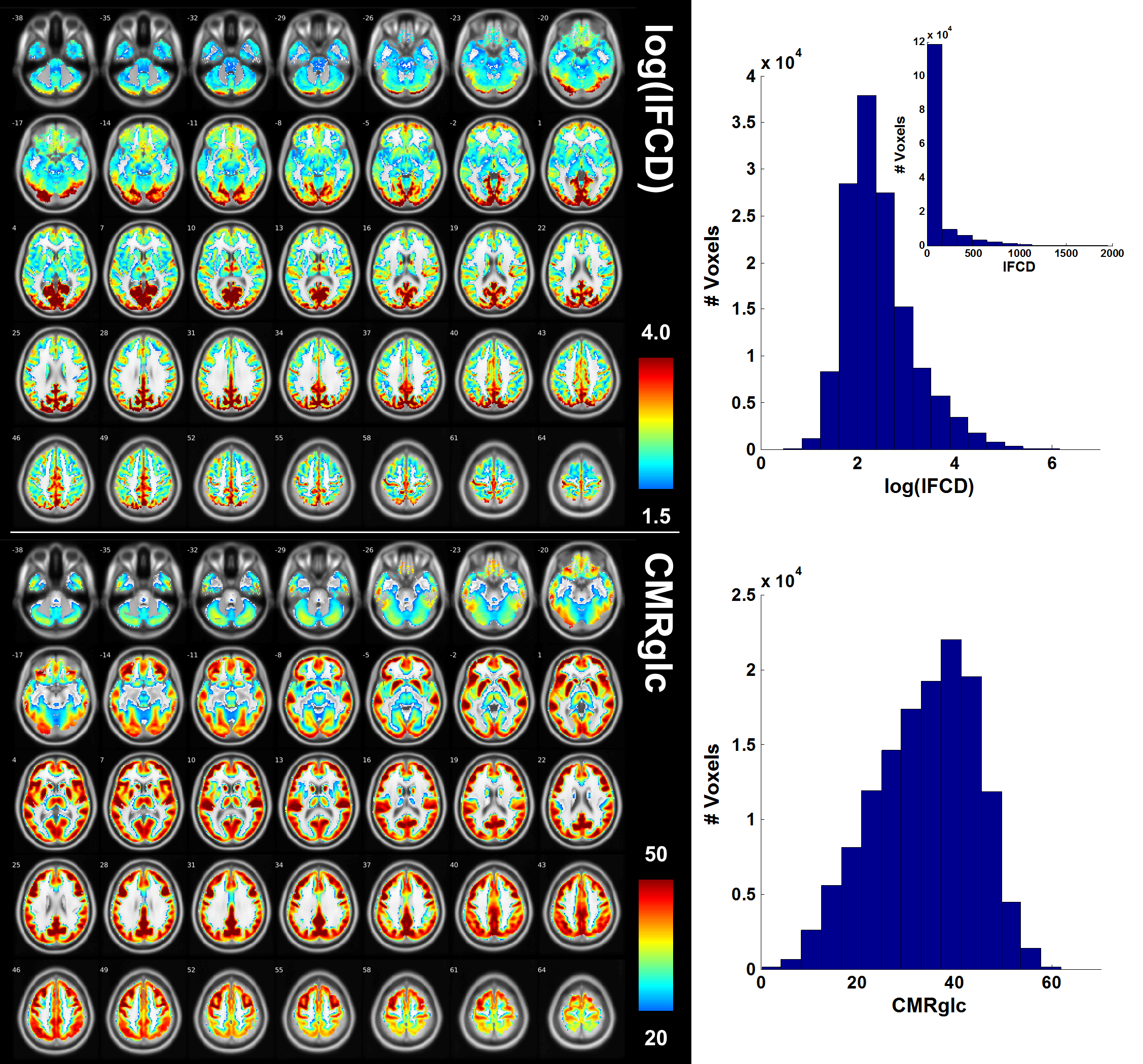
**

**Supplementary Figure 2.** Regional distribution of log(lFCD) (top row) and CMRglc (bottom row) in cohort-1 (*n* = 28), along with their voxelwise histograms (15 bins) in gray matter (averaged across subjects). The histogram of lFCD (without log transformation) is also shown (in top right corner of the log(lFCD) histogram) with a heavily skewed distribution (skewness = 3.22). In comparison, log(lFCD) had lower skewness (skewness = 0.90), closer to a normal distribution. The MNI *z*-coordinate is displayed next to each slice.

**
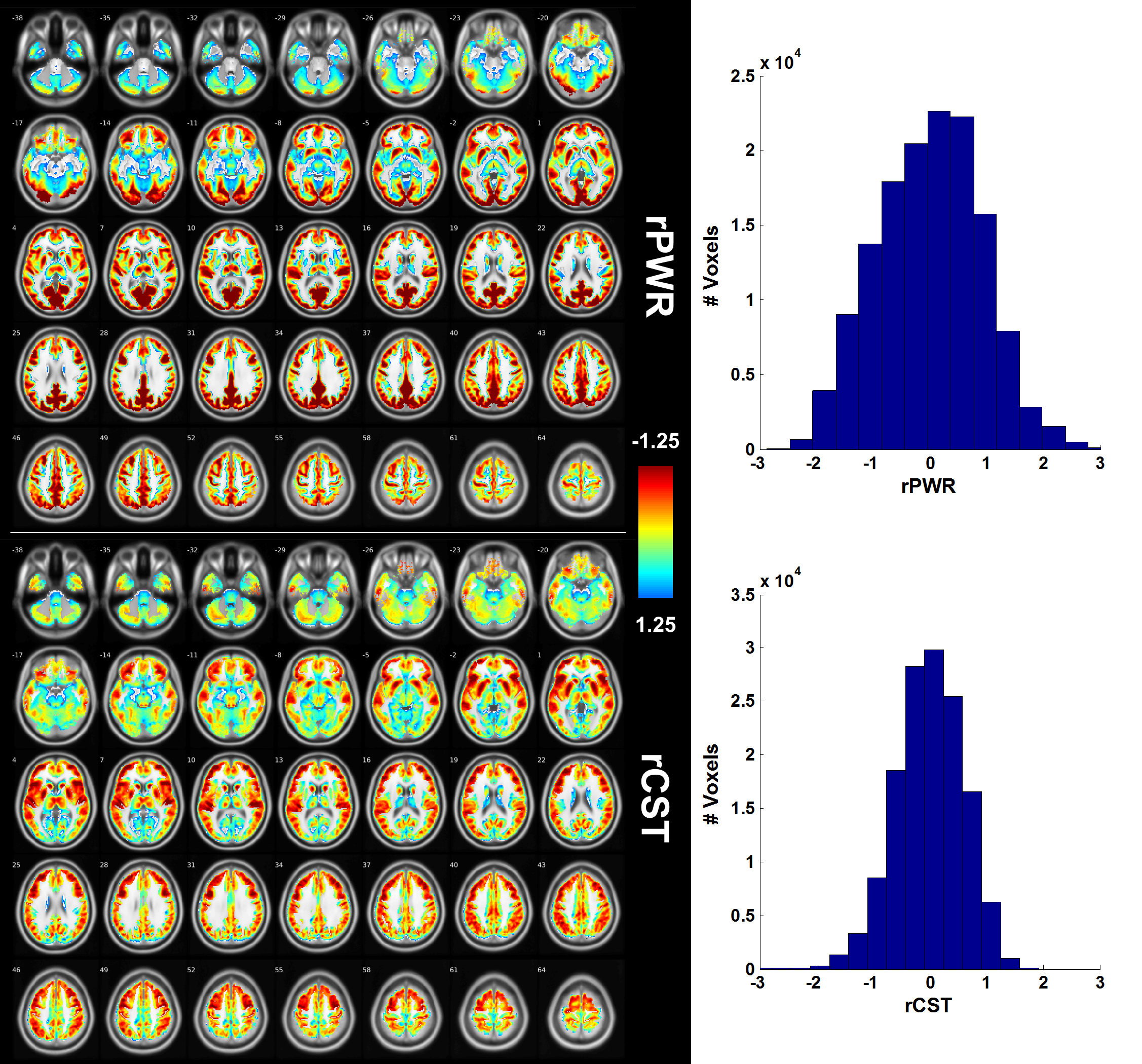
**

**Supplementary Figure 3.** Regional distribution of rPWR (top row) and rCST (bottom row) in cohort-1 (n = 28), along with their voxelwise histograms (15 bins) in the gray matter (averaged across subjects). The MNI *z*-coordinate is displayed next to each slice.

**Supplementary Figure 4.** Calinski-Harabasz clustering evaluation criterion[^21^](#_ENREF_21). Clustering was performed on gray matter voxels based on across-subject average (cohort-1, *n* = 28) of rPWR and rCST measures (2 × *n* data points; *n* = 139267 voxels). While cluster count = 2 achieved the highest criteria score, there was a local maximum at 4 clusters. These 4 clusters were 100% reproducible with repeated execution (*n* = 100) of *k*-means algorithm (with 1000 maximum interactions for convergence of each *k*-means execution).

**
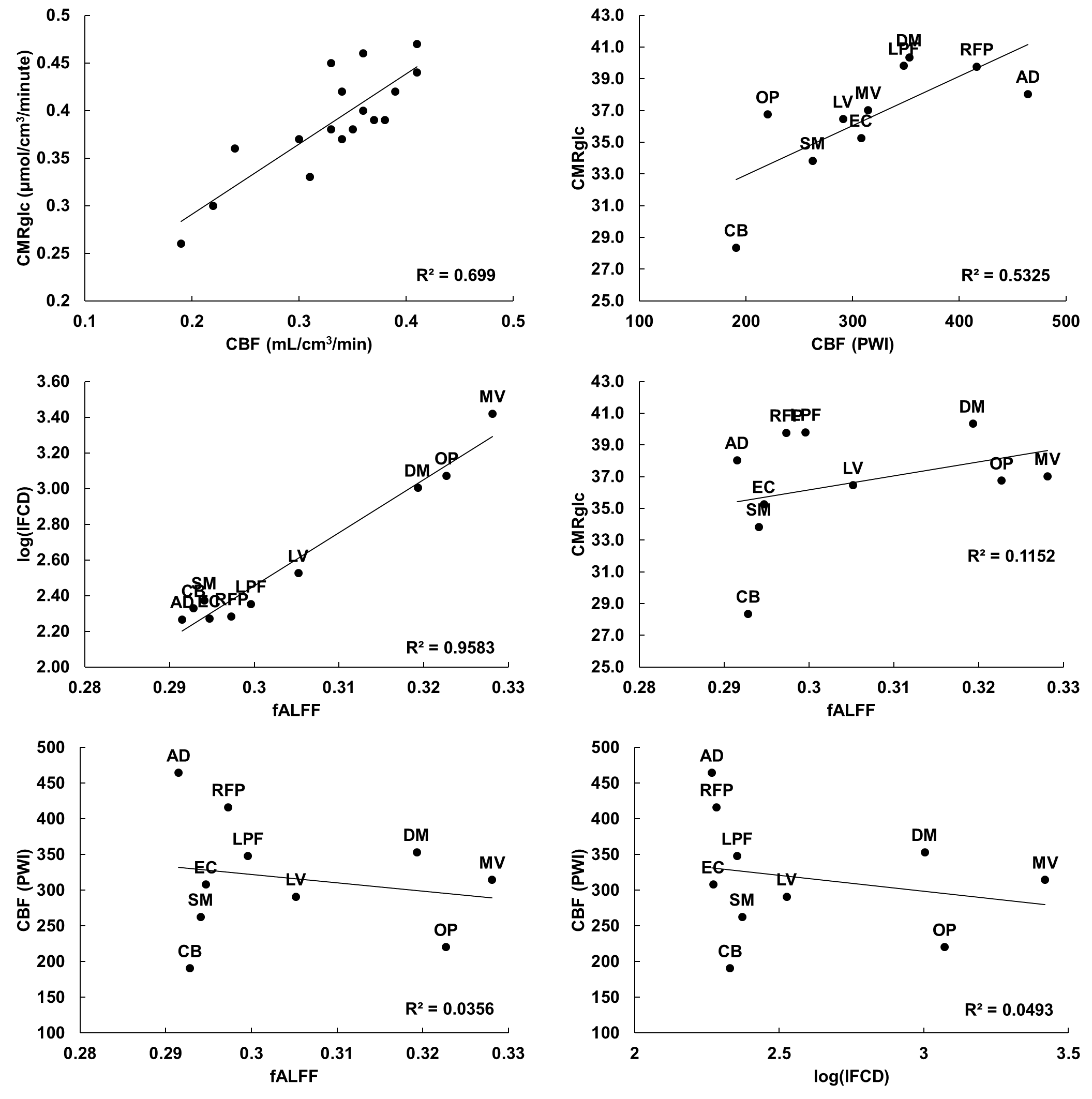
**

**e**

**f**

**c**

**d**

**b**

**a**

**Supplementary Figure 5.** Associations between CMRglc, CBF (PWI), lFCD, and fALFF. (**a**) Correlation between CBF and CMRglc of gray matter structures reported in an independent publication[^22^](#_ENREF_22) (*r*(8) = 0.84, *p* < 0.001). (**b**) Correlation between CBF (PWI) and CMRglc of 10 brain networks in cohort-1 (*r*(8) = 0.73, *p* = 0.017). (**c**) Correlation between fALFF and log(lFCD) in cohort-1 (*r*(8) = 0.98, *p* < 0.001). (**d**) Correlation between fALFF and CMRglc in cohort-1 (*r*(8) = 0.34, *p* = 0.34). (**e**) Correlation between fALFF and CBF (PWI) in cohort-1 (*r*(8) = -0.19, *p* = 0.60). (**f**) Correlation between log(lFCD) and CBF (PWI) in cohort-1 (*r*(8) = -0.22, *p* = 0.54). Each point represents across-subject average for medial visual (MV), occipital pole (OP), lateral visual (LV), default mode (DM), cerebellum (CB), sensorimotor (SM), auditory (AD), executive control (EC), right and left frontoparietal (RFP & LFP) networks.


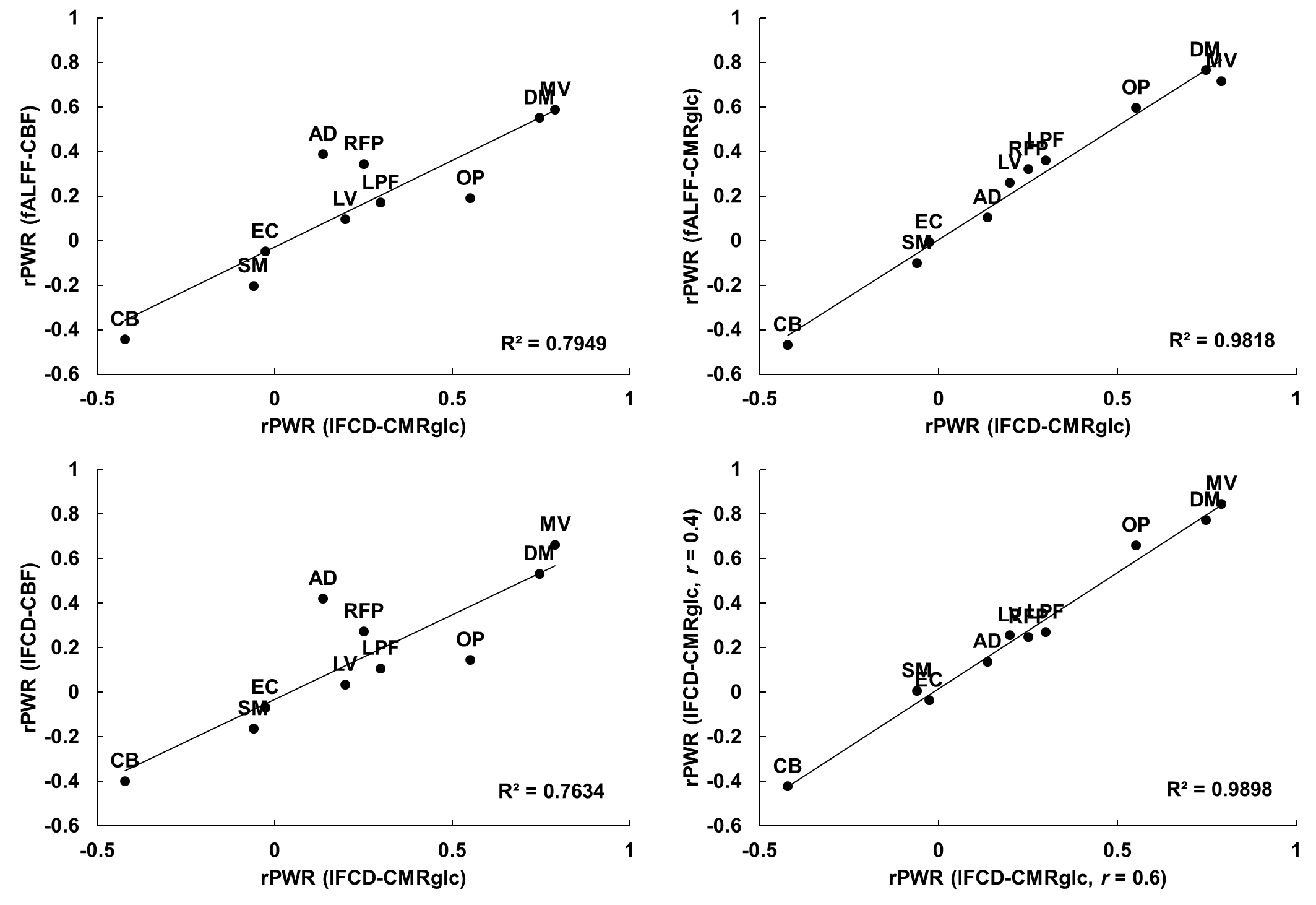


**d**

**c**

**b**

**a**

**Supplementary Figure 6.** Generalizability of rPWR in cohort-1. (**a**) Correlation between lFCD-CMRglc based rPWR and fALFF-CBF based rPWR (*r*(8) = 0.89, *p* < 0.001). (**b**) Correlation between lFCD-CMRglc based rPWR and fALFF-CMRglc based rPWR (*r*(8) = 0.99, *p* < 0.001). (**c**) Correlation between lFCD-CMRglc based rPWR and lFCD-CBF based rPWR (*r*(8) = 0.87, *p* < 0.001). (**d**) Correlation between lFCD-CMRglc based rPWR with *r* = 0.6 lFCD threshold (default) and lFCD-CMRglc based rPWR with *r* = 0.4 lFCD threshold (*r*(8) = 0.99, *p* < 0.001). Each point represents across-subject average for medial visual (MV), occipital pole (OP), lateral visual (LV), default mode (DM), cerebellum (CB), sensorimotor (SM), auditory (AD), executive control (EC), right and left frontoparietal (RFP & LFP) networks.


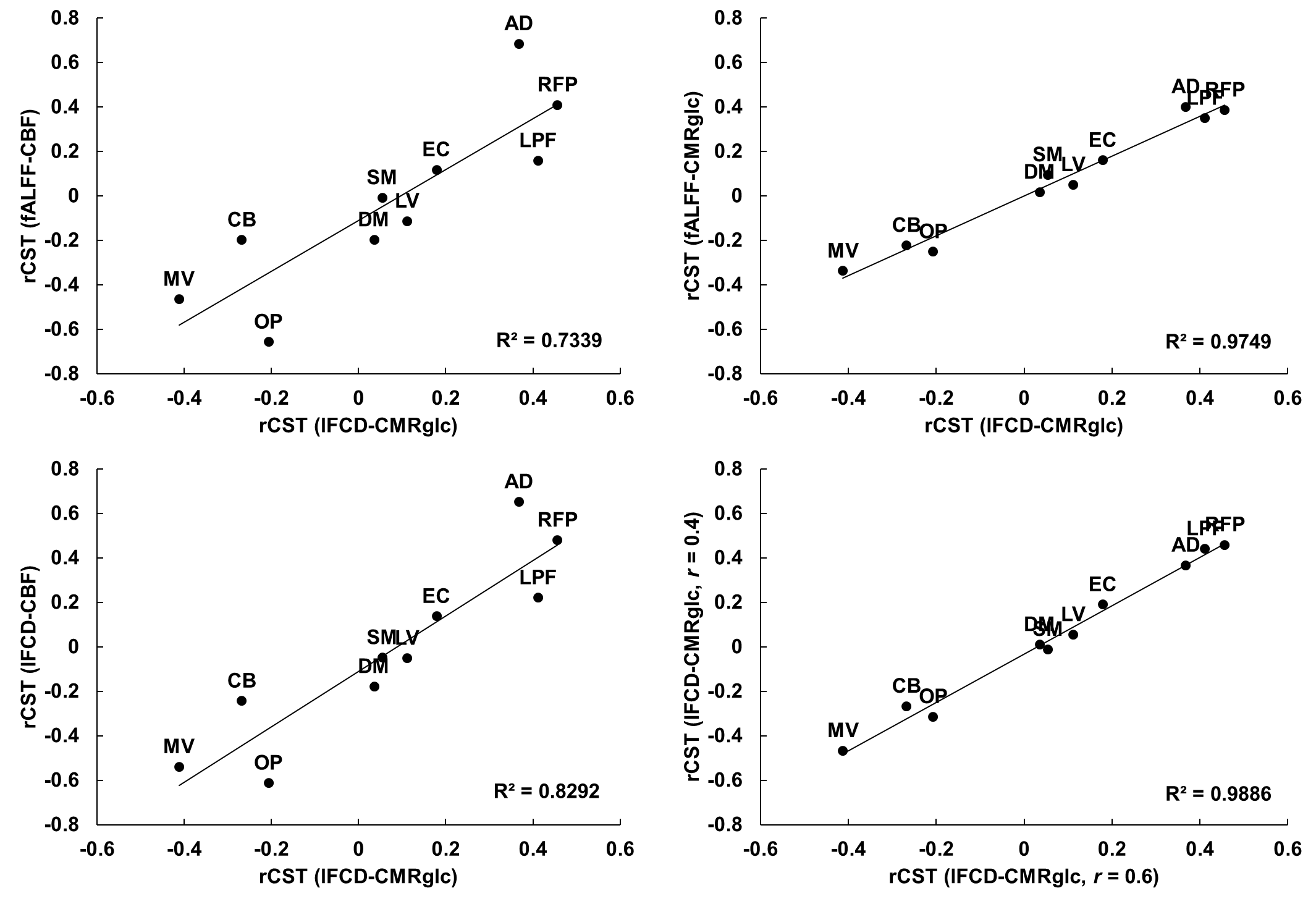


**d**

**c**

**b**

**a**

**Supplementary Figure 7.** Generalizability of rCST in cohort-1. (**a**) Correlation between lFCD-CMRglc based rCST and fALFF-CBF based rCST (*r*(8) = 0.89, *p* < 0.001). (**b**) Correlation between lFCD-CMRglc based rCST and fALFF-CMRglc based rCST (*r*(8) = 0.99, *p* < 0.001). (**c**) Correlation between lFCD-CMRglc based rCST and lFCD-CBF based rCST (*r*(8) = 0.87, *p* < 0.001). (**d**) Correlation between lFCD-CMRglc based rCST with *r* = 0.6 lFCD threshold (default) and lFCD-CMRglc based rCST with *r* = 0.4 lFCD threshold (*r*(8) = 0.99, *p* < 0.001). Each point represents across-subject average for medial visual (MV), occipital pole (OP), lateral visual (LV), default mode (DM), cerebellum (CB), sensorimotor (SM), auditory (AD), executive control (EC), right and left frontoparietal (RFP & LFP) networks.


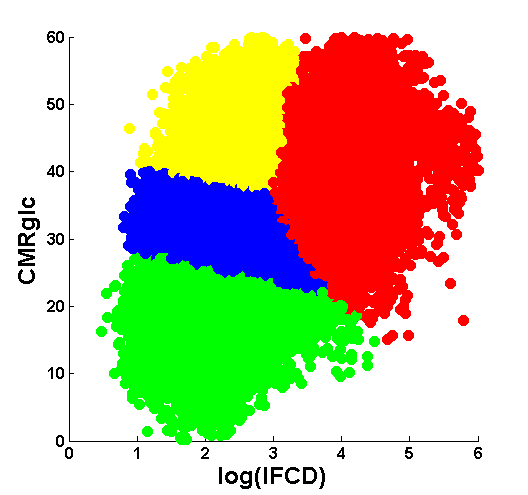

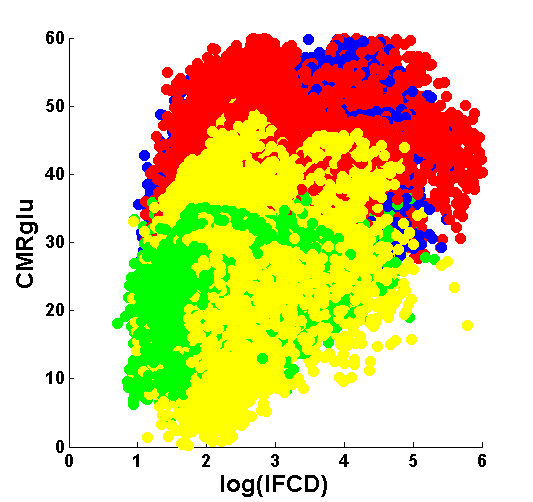


**b**

**a**

**Supplementary Figure 8.** Effect of tSNR on segmentation (cohort-1, n = 28). (**a**) Original segmentation (4 clusters) of the brain based on rPWR and rCST computed with lFCD-CMRglc then projected back into the lFCD-CMRglc space (also shown in Fig. 3b). (**b**) Segmentation (4 clusters) of brain based on rPWR and rCST calculated for tSNR_fMRI_-tSNR_FDG-PET_ then projected back into the lFCD-CMRglc space. Colors are arbitrary.


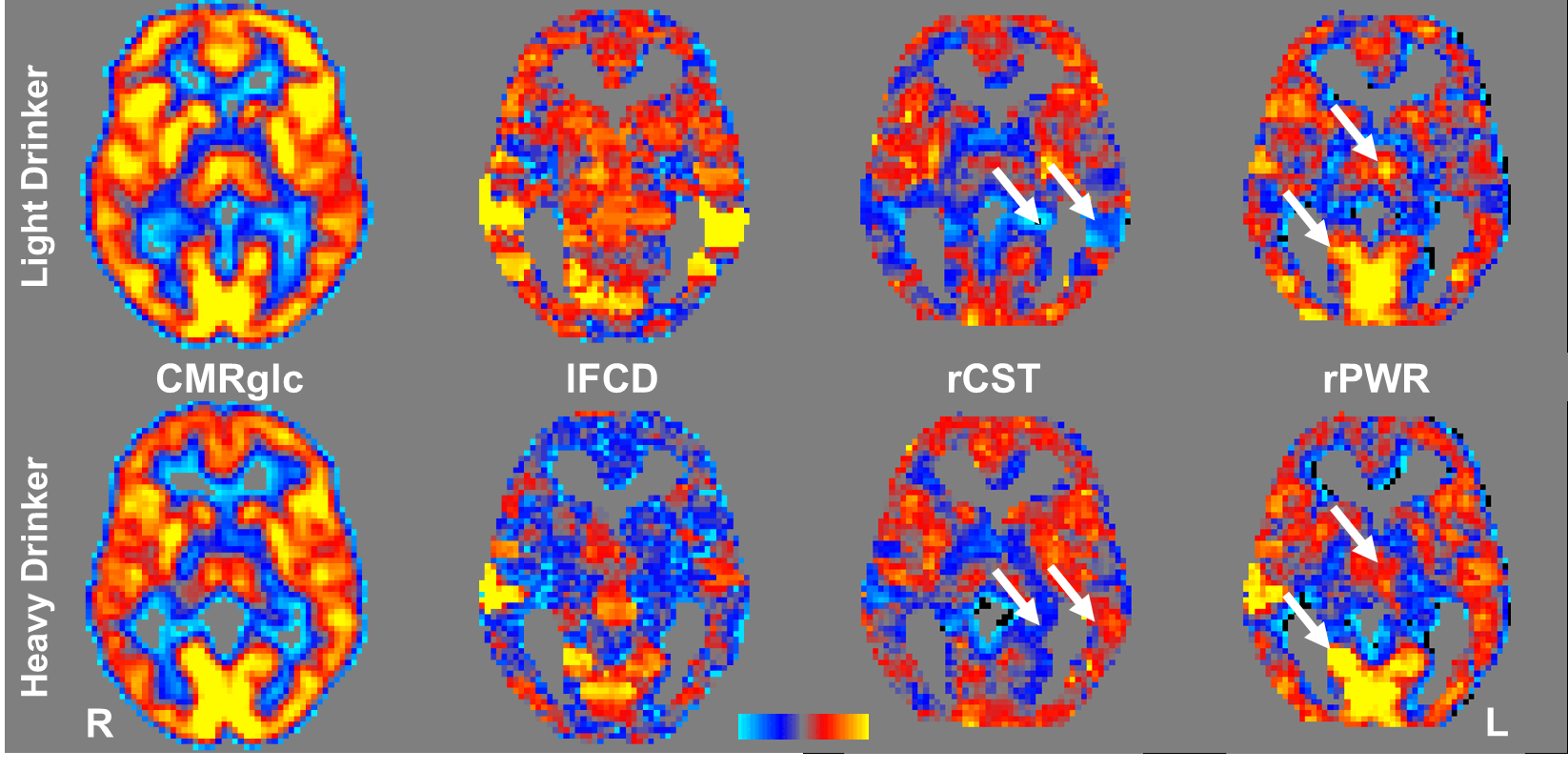


**Supplementary Figure 9.** Maps of CMRglc, lFCD, rCST, and rPWR for a representative light drinker (LD) (top row) and a representative heavy drinker (HD) (bottom row) of cohort-2 for the same cross section of the brain in the MNI space (*z* = 0 mm). The same color scale was used for the top and bottom images in each column. The arrows highlight some of the group differences in rCST and rPWR reported in Supplementary Tables 15, 16. Images are shown in radiological convention (right is left). The color scale blue to yellow indicate low to high values in the maps, respectively.


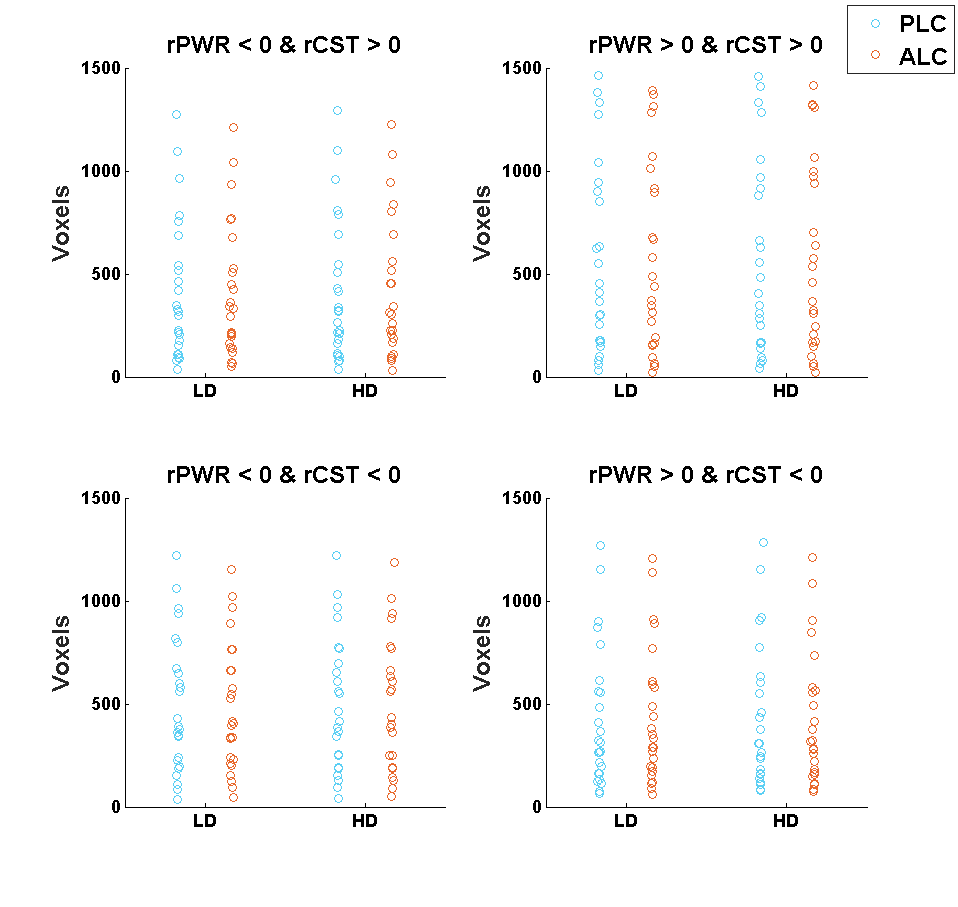


**a**

**b**

**Supplementary Figure 10.** Raw data used to generate bar plots in Fig. 4g. (**a**) Two-dimensional histograms of rPWR versus rCST measures for light (LD) and heavy (HD) drinkers under placebo (PLC) and alcohol (ALC) conditions. (**b**) Each data point shows the number of voxels (aggregated across subjects and normalized by number of subjects per group) falling into one of 25 (5 × 5) partitions (divided by the 0 axes) of rPWR and rCST that were used to estimate the 2-dimensional histograms shown in (**a**). Regional rPWR and rCST were computed from whole brain normalized *log*(lFCD) and CMRglc, thus they had zero-mean and unit variance for each participant and condition (see Fig. 4g).

**Supplementary Table 1.** One sample *t*-test on rPWR (*p* < 10^-7^, uncorrected, *p*_FWE_ < 0.01, |t| > 6, corrected at voxel-level, *k* > 400). Coordinates are in MNI space (voxel size = 2-mm isotropic).

| **Region(s)** | **L/R** | **Contrast** | **Brodmann area(s)** | **Cluster size** | **Peak coordinates (x, y, z)_mm_** | | | **Peak *t*-value** | |
| --- | --- | --- | --- | --- | --- | --- | --- | --- | --- |
| **Limbic Lobe**  ***Posterior Cingulate***  **Occipital Lobe**  ***Calcarine***  ***Cuneus***  ***Lingual Gyrus***  ***Middle Occipital Gyrus***  **Parietal Lobe**  ***Angular Gyrus***  ***Precuneus***  **Temporal Lobe**  ***Middle Temporal Gyrus*** | L/R  L/R  L/R  L/R | rPWR > 0 | 7, 17, 18, 19, 23, 30, 31, 39 | 9677 | -1 | -45 | 33 | | 23.57 |
| **Frontal Lobe**  ***Inferior Frontal Gyrus***  ***Middle Frontal Gyrus***  ***Precentral Gyrus***  ***Superior Frontal Gyrus***  **Insula**  **Parietal Lobe**  ***Inferior Parietal Lobule***  **Temporal Lobe**  ***Superior Temporal Gyrus*** | R  R  R  R | rPWR > 0 | 9, 10, 13, 11, 41, 46 | 2323 | 25 | 58 | -1 | | 18.48 |
| **Temporal Lobe**  ***Superior Temporal Gyrus***  **Insula**  **Parietal Lobe**  ***Inferior Parietal Lobule*** | L  L  L | rPWR > 0 | 13, 40, 41 | 698 | -41 | -29 | 11 | | 16.38 |
| **Frontal Lobe**  ***Inferior Frontal Gyrus***  ***Middle Frontal Gyrus***  ***Precentral Gyrus***  ***Superior Frontal Gyrus***  **Insula** | L  L | rPWR > 0 | 9, 10, 11, 47, 46 | 2099 | -33 | 36 | -13 | | 18.93 |
| **Putamen**  **Caudate** | L | rPWR > 0 | - | 584 | -27 | 6 | 1 | | 24.23 |
| **Putamen**  **Caudate** | R | rPWR > 0 | - | 522 | 27 | 2 | 5 | | 21.87 |
| **Cerebellum (Anterior and Posterior Lobe)**  ***Cerebellar Tonsil***  ***Culmen***  ***Declive***  **Frontal lobe**  ***Medial Frontal Gyrus***  ***Inferior Frontal Gyrus***  ***Precentral Gyrus***  **Insula**  **Limbic Lobe**  ***Cingulate Gyrus***  ***Hippocampus***  ***Parahippocampal Gyrus***  **Midbrain**  **Medulla**  **Pons**  **Thalamus***  ***Ventral Anterior Nucleus***  ***Ventral Lateral Nucleus***  **Temporal Lobe**  ***Fusiform Gyrus***  ***Superior Temporal Gyrus***  ***Inferior Temporal Gyrus*** | L/R  L/R  L/R  L/R  L/R  L/R  L/R  L/R  L/R | rPWR < 0 | 13, 20, 21, 24, 36, 38 | 34587 | -11 | -43 | 9 | | -60.86 |

***Bilateral medial dorsal nucleus of the thalamus also had significantly high rPWR (*k* = 184, *p*_FWE_ < 0.01, cluster-size corrected).**

**Supplementary Table 2.** One sample *t*-test on rCST (*p* < 10^-7^, uncorrected, *p*_FWE_ < 0.01, |t| > 6, corrected at voxel-level, *k* > 400). Coordinates are in MNI space (voxel size = 2-mm isotropic).

| **Region(s)** | **L/R** | **Contrast** | **Brodmann area(s)** | **Cluster size** | **Peak coordinates (x, y, z)_mm_** | | | **Peak *t*-value** | |
| --- | --- | --- | --- | --- | --- | --- | --- | --- | --- |
| **Frontal Lobe**  ***Inferior Frontal Gyrus***  ***Middle Frontal Gyrus***  ***Precentral Gyrus***  ***Superior Frontal Gyrus***  **Insula**  **Parietal Lobe**  ***Inferior Parietal Lobule***  ***Postcentral Gyrus***  **Putamen**  **Temporal Lobe**  ***Middle Temporal Gyrus***  ***Superior Temporal Gyrus*** | L  L  L  L  L | rCST > 0 | 6, 9, 10, 13, 40, 47 | 8671 | -43 | -53 | 47 | | 28.30 |
| **Frontal Lobe**  ***Inferior Frontal Gyrus***  ***Middle Frontal Gyrus***  ***Precentral Gyrus***  ***Superior Frontal Gyrus***  **Insula**  **Putamen**  **Temporal Lobe**  ***Superior Temporal Gyrus***  ***Middle Temporal Gyrus*** | R  R  R  R | rCST > 0 | 6, 9, 10, 22, 13, 47 | 5632 | 37 | -5 | 53 | | 28.76 |
| **Caudate**  **Cerebellum (Anterior and Posterior Lobe)**  ***Culmen***  ***Declive***  **Limbic Lobe**  ***Amygdala***  ***Anterior Cingulate***  ***Hippocampus***  ***Parahippocampal Gyrus***  **Midbrain**  **Occipital Lobe**  ***Fusiform Gyrus***  ***Lingual Gyrus***  **Pons**  **Temporal Lobe**  ***Superior Temporal Gyrus***  **Thalamus***  ***Ventral Anterior Nucleus***  ***Ventral Lateral Nucleus*** | L/R  L/R  L/R  L/R  L/R  L/R  L/R  L/R | rCST < 0 | 25, 28, 34, 38 | 15316 | -23 | -31 | -40 | | 42.85 |

***Bilateral medial dorsal nucleus of the thalamus also had significantly higher rCST (*k* = 199, *p*_FWE_ < 0.01, cluster-size corrected).**

**Supplementary Table 3.** Paired *t*-test between rCST and rPWR (*p* < 10^-7^, uncorrected, *p*_FWE_ < 0.01, |t| > 6, corrected at voxel-level, *k* > 400). Coordinates are in MNI space (voxel size: 2-mm isotropic).

| **Region(s)** | **L/R** | **Contrast** | **Brodmann area(s)** | **Cluster size** | **Peak coordinates (x, y, z)_mm_** | | | **Peak *t*-value** | |
| --- | --- | --- | --- | --- | --- | --- | --- | --- | --- |
| **Cerebellum (Anterior and Posterior Lobes)**  ***Culmen***  ***Devlive***  **Frontal Lobe**  ***Inferior Frontal Gyrus***  **Limbic Lobe**  ***Amygdala***  ***Hippocampus***  ***Parahippocampal Gyrus***  **Globus Pallidus**  **Insula**  **Medulla**  **Midbrain**  **Pons**  **Putamen**  **Temporal Lobe**  ***Fusiform Gyrus***  ***Inferior Temporal Gyrus***  ***Middle Temporal Gyrus***  ***Superior Temporal Gyrus***  **Thalamus***  ***Ventral Anterior Nucleus***  ***Ventral Lateral Nucleus*** | L/R  L/R  L/R  L/R  L/R  L/R  L/R  L/R  L/R  L/R  L/R | **rCST > rPWR** | 13, 20, 21, 22, 28, 36, 38 | 8671 | -43 | -53 | 47 | | 28.30 |
| **Limbic Lobe**  ***Middle Cingulate***  **Frontal Lobe**  ***Medial Frontal Gyrus***  ***Precentral Gyrus***  ***Superior Frontal Gyrus***  **Parietal Lobe**  ***Inferior Parietal Lobule***  ***Postcentral Gyrus***  ***Precuneus*** | L  L  L | **rCST > rPWR** | 6, 4, 7 | 817 | -39 | -13 | 35 | | 17.24 |
| **Limbic Lobe**  ***Middle Cingulate***  **Frontal Lobe**  ***Medial Frontal Gyrus***  ***Precentral Gyrus*** | R  R | **rCST > rPWR** | 6 | 438 | 9 | -19 | 63 | | 14.69 |
| **Limbic Lobe**  ***Posterior Cingulate***  **Occipital Lobe**  ***Calcarine***  ***Cuneus***  ***Middle Occipital Gyrus***  ***Lingual Gyrus***  **Parietal Lobe**  ***Angular Gyrus***  ***Precuneus***  **Temporal Lobe**  ***Middle Temporal Gyrus*** | L/R  L/R  L/R  L/R  L/R | **rPWR > rCST** | 7, 18, 17, 19, 30, 31 | 8173 | 9 | -71 | 15 | | -18.41 |

***Medial dorsal nucleus of the thalamus had significantly higher rPWR than rCST (*k* = 73, *p*_FWE_ < 0.01, cluster-size corrected).**

**Supplementary Table 4.** Anatomy of the sensorimotor cluster as identified by the *k*-means clustering approach (*k* > 400). In addition to sensorimotor areas, this cluster also included a range of regions such as limbic and visual areas.

| **Region(s)** | **L/R** | **Brodmann area(s)** | **Cluster size** | **Cluster Mean rPWR** | **Cluster Mean rCST** |
| --- | --- | --- | --- | --- | --- |
| **Caudate**  **Cerebellum (Anterior and Posterior Lobe)**  ***Culmen***  ***Declive***  ***Uvula***  **Frontal Lobe**  ***Inferior Frontal Gyrus***  ***Medial Frontal Gyrus***  ***Middle Frontal Gyrus***  ***Paracentral Lobule***  ***Precentral Gyrus***  ***Superior Frontal Gyrus***  **Insula**  **Limbic Lobe**  ***Anterior Cingulate***  ***Parahippocampal Gyrus***  **Occipital Lobe**  ***Cuneus***  ***Lingual Gyrus***  ***Middle Occipital Gyrus***  **Parietal Lobe**  ***Inferior Parietal Lobule***  ***Postcentral Gyrus***  ***Precuneus***  ***Supramarginal Gyrus***  **Putamen**  **Temporal Lobe**  ***Fusiform Gyrus***  ***Inferior Temporal Gyrus***  ***Middle Temporal Gyrus***  ***Superior Temporal Gyrus*** | L/R  L/R  L/R  L/R  L/R  L/R  L/R  L/R  L/R | 3, 4, 6, 7, 10, 11, 13, 20, 21, 22, 24, 32, 37, 38, 40, 47, | 44185 | -0.27 | 0.01 |

**Supplementary Table 5.** Anatomy of the cerebellar-limbic cluster as identified by the *k*-means clustering approach (*k* > 400). In addition to cerebellar-limbic areas, this cluster also included a range of regions such as frontal and temporal areas.

| **Region(s)** | **L/R** | **Brodmann area(s)** | **Cluster size** | **Cluster Mean rPWR** | **Cluster Mean rCST** |
| --- | --- | --- | --- | --- | --- |
| **Caudate**  **Cerebellum (Anterior and Posterior Lobe)**  ***Culmen***  ***Declive***  **Frontal Lobe**  ***Medial Frontal Gyrus***  ***Inferior Frontal Gyrus***  **Globus Pallidus**  **Insula**  **Limbic Lobe**  ***Amygdala***  ***Anterior Cingulate***  ***Hippocampus_***  ***Parahippocampal Gyrus***  **Medulla**  **Midbrain**  **Occipital Lobe**  ***Cuneus***  ***Lingual Gyrus***  **Pons**  **Putamen**  **Thalamus**  ***Ventral Anterior Nucleus***  ***Ventral Lateral Nucleus***  **Temporal Lobe**  ***Fusiform Gyrus***  ***Inferior Temporal Gyrus***  ***Middle Temporal Gyrus***  ***Superior Temporal Gyrus*** | L/R  L/R  L/R  L/R  L/R  L/R  L/R  L/R  L/R  L/R  L/R  L/R  L/R | 13, 20, 21, 24, 28, 34, 36, 38 | 27780 | -1.25 | -0.59 |
| **Frontal Lobe**  ***Medial Frontal Gyrus***  **Limbic Lobe**  ***Middle Cingulate***  **Parietal Lobe**  ***Precuneus*** | L  L  L | 6, 31 | 765 | -1.12 | -0.33 |

**Supplementary Table 6.** Anatomy of the visual cluster as identified by the *k*-means clustering approach (*k* > 400). In addition to visual areas, this cluster also included a range of regions such as frontal and parietal areas. Medial dorsal nucleus of the thalamus was also identified as a part of this network (*k* = 159).

| **Region(s)** | **L/R** | **Brodmann area(s)** | **Cluster size** | **Cluster Mean rPWR** | **Cluster Mean rCST** |
| --- | --- | --- | --- | --- | --- |
| **Cerebellum (Posterior Lobe)**  ***Declive***  **Limbic Lobe**  ***Middle Cingulate***  ***Posterior Cingulate***  **Occipital Lobe**  ***Cuneus***  ***Inferior Occipital Gyrus***  ***Lingual Gyrus***  ***Middle Occipital Gyrus***  ***Superior Occipital Gyrus***  **Parietal Lobe**  ***Angular Gyrus***  ***Inferior Parietal Lobule***  ***Precuneus***  ***Superior Parietal Lobule***  **Temporal Lobe**  ***Fusiform Gyrus***  ***Inferior Temporal Gyrus***  ***Middle Temporal Gyrus*** | L/R  L/R  L/R  L/R  L/R | 5, 6, 7, 17, 18, 19, 23, 30, 31, 37, 39 | 18628 | 1.25 | -0.07 |
| **Frontal Lobe**  ***Medial Frontal Gyrus***  ***Superior Frontal Gyrus*** | L/R | 9, 10 | 476 | 0.88 | -0.07 |
| **Frontal Lobe**  ***Precentral Gyrus***  **Insula**  **Parietal Lobe**  ***Inferior Parietal Lobule***  ***Postcentral Gyrus*** | R  R  R | 4, 6, 13, 40 | 652 | 0.92 | -0.21 |
| **Frontal Lobe**  ***Precentral Gyrus***  **Parietal Lobe**  ***Inferior Parietal Lobule*** | L  L | 3, 4, 6 | 457 | 1.01 | -0.19 |

**Supplementary Table 7.** Anatomy of the frontoparietal cluster as identified by the *k*-means clustering approach (*k* > 400). In addition to frontoparietal areas, this cluster also included a range of regions such as occipital and temporal areas.

| **Region(s)** | **L/R** | **Brodmann area(s)** | **Cluster size** | **Cluster Mean rPWR** | **Cluster Mean rCST** |
| --- | --- | --- | --- | --- | --- |
| **Caudate**  **Frontal Lobe**  ***Medial Frontal Gyrus***  ***Middle Frontal Gyrus***  ***Inferior Frontal Gyrus***  ***Precentral Gyrus***  ***Superior Frontal Gyrus***  **Insula**  **Limbic Lobe**  ***Anterior Cingulate***  **Occipital Lobe**  ***Cuneus***  ***Middle Occipital Gyrus***  **Parietal Lobe**  ***Angular Gyrus***  ***Inferior Parietal Lobule***  ***Postcentral Gyrus***  ***Precuneus***  ***Supramarginal Gyrus***  ***Superior Parietal Lobule***  **Putamen**  **Temporal Lobe**  ***Fusiform Gyrus***  ***Inferior Temporal Gyrus***  ***Middle Temporal Gyrus***  ***Superior Temporal Gyrus***  ***Transverse Temporal Gyrus*** | L  L/R  L/R  L/R  L/R  L/R  L  L/R | 3, 4, 6, 7, 8, 9, 10, 11, 13, 19, 21, 22, 24, 31, 32, 37, 39, 40, 41, 44, 45, 46, 47 | 39314 | 0.61 | 0.61 |
| **Caudate**  **Pallidum**  **Putamen** | R  R  R | - | 873 | 0.64 | 0.76 |
| **Thalamus**  ***Medial Dorsal Nucleus***  ***Ventral Lateral Nucleus*** | L/R |  | 430 | 0.46 | 0.50 |

**Supplementary Table 8.** Across-subject mean (M) and standard deviation (SD) of lFCD, CMRglc, rCST, and rPWR highlighting individual differences (cohort-1, *n* = 28).

| **Region** | **log(lFCD)**  **(M, SD)** | | **CMRglc**  **(M, SD)** | | **rCST**  **(M, SD)** | | **rPWR**  **(M, SD)** | |
| --- | --- | --- | --- | --- | --- | --- | --- | --- |
| **bankssts** | 2.46 | 0.34 | 40.36 | 5.04 | 0.40 | 0.15 | 0.40 | 0.20 |
| **caudalanteriorcingulate** | 2.52 | 0.33 | 35.12 | 5.78 | 0.02 | 0.22 | 0.12 | 0.20 |
| **caudalmiddlefrontal** | 2.47 | 0.36 | 44.30 | 6.81 | 0.60 | 0.22 | 0.66 | 0.26 |
| **cuneus** | 4.24 | 0.93 | 44.96 | 7.16 | -0.44 | 0.29 | 1.78 | 0.44 |
| **entorhinal** | 1.89 | 0.24 | 19.21 | 3.22 | -0.56 | 0.18 | -1.25 | 0.16 |
| **fusiform** | 2.48 | 0.36 | 31.83 | 4.22 | -0.14 | 0.13 | -0.12 | 0.14 |
| **inferiorparietal** | 3.01 | 0.38 | 41.42 | 5.72 | 0.09 | 0.16 | 0.82 | 0.20 |
| **inferiortemporal** | 2.31 | 0.23 | 34.04 | 5.01 | 0.08 | 0.17 | -0.08 | 0.13 |
| **isthmuscingulate** | 3.49 | 0.52 | 37.66 | 6.49 | -0.44 | 0.20 | 0.88 | 0.23 |
| **lateraloccipital** | 3.56 | 0.67 | 39.99 | 5.10 | -0.31 | 0.21 | 1.06 | 0.27 |
| **lateralorbitofrontal** | 2.28 | 0.16 | 37.65 | 5.88 | 0.31 | 0.22 | 0.13 | 0.13 |
| **lingual** | 3.68 | 0.82 | 36.01 | 4.73 | -0.62 | 0.29 | 0.88 | 0.31 |
| **medialorbitofrontal** | 2.53 | 0.21 | 34.83 | 5.51 | -0.01 | 0.19 | 0.12 | 0.19 |
| **middletemporal** | 2.33 | 0.19 | 36.69 | 5.52 | 0.22 | 0.18 | 0.11 | 0.14 |
| **parahippocampal** | 2.12 | 0.25 | 22.78 | 3.28 | -0.47 | 0.10 | -0.90 | 0.14 |
| **paracentral** | 2.86 | 0.62 | 33.23 | 4.26 | -0.28 | 0.28 | 0.22 | 0.34 |
| **parsopercularis** | 2.40 | 0.27 | 45.76 | 7.00 | 0.74 | 0.14 | 0.69 | 0.23 |
| **parsorbitalis** | 2.54 | 0.30 | 41.66 | 7.52 | 0.39 | 0.29 | 0.53 | 0.24 |
| **parstriangularis** | 2.42 | 0.32 | 42.48 | 6.93 | 0.53 | 0.23 | 0.50 | 0.22 |
| **pericalcarine** | 4.71 | 1.03 | 39.98 | 6.96 | -1.06 | 0.47 | 1.79 | 0.45 |
| **postcentral** | 2.60 | 0.39 | 35.73 | 4.51 | 0.03 | 0.20 | 0.20 | 0.22 |
| **posteriorcingulate** | 2.92 | 0.35 | 40.62 | 5.80 | 0.10 | 0.15 | 0.72 | 0.24 |
| **precentral** | 2.49 | 0.35 | 38.76 | 4.99 | 0.27 | 0.18 | 0.32 | 0.19 |
| **precuneus** | 3.54 | 0.52 | 42.47 | 6.00 | -0.17 | 0.16 | 1.21 | 0.13 |
| **rostralanteriorcingulate** | 2.39 | 0.20 | 35.24 | 5.27 | 0.10 | 0.19 | 0.05 | 0.16 |
| **rostralmiddlefrontal** | 2.54 | 0.27 | 45.09 | 6.99 | 0.61 | 0.11 | 0.74 | 0.19 |
| **superiorfrontal** | 2.46 | 0.27 | 39.53 | 5.95 | 0.33 | 0.09 | 0.35 | 0.18 |
| **superiorparietal** | 3.29 | 0.64 | 37.45 | 4.93 | -0.30 | 0.21 | 0.73 | 0.23 |
| **superiortemporal** | 2.18 | 0.23 | 34.54 | 4.79 | 0.21 | 0.14 | -0.13 | 0.14 |
| **supramarginal** | 2.63 | 0.27 | 39.05 | 5.07 | 0.20 | 0.14 | 0.44 | 0.16 |
| **frontalpole** | 2.81 | 0.54 | 35.79 | 6.37 | -0.16 | 0.40 | 0.36 | 0.39 |
| **temporalpole** | 1.87 | 0.22 | 19.64 | 3.82 | -0.53 | 0.19 | -1.24 | 0.18 |
| **transversetemporal** | 2.49 | 0.42 | 48.42 | 6.82 | 0.87 | 0.19 | 0.90 | 0.28 |
| **insula** | 2.15 | 0.16 | 32.01 | 4.82 | 0.07 | 0.12 | -0.31 | 0.12 |

**Supplementary Table 9.** Columns 2–5 show correlations between regional cortical thickness and regional lFCD, CMRglc, rCST, and rPWR across subjects (cohort-1, *n* = 28). Regions are sorted by their mean cortical thickness (last column) (**p*< 0.05, ***p*< 0.05 Bonferroni). Mean and SD and significance levels are reported for the Fisher’s z-transformed correlations across regions.

| **Region** | **lFCD** | **CMRglc** | **rCST** | **rPWR** | **Mean Thickness (mm)** |
| --- | --- | --- | --- | --- | --- |
| **pericalcarine** | *0.276* | *-0.176* | *-0.294* | *0.027* | 1.62 |
| **cuneus** | *0.239* | *0.109* | *-0.194* | *0.029* | 1.87 |
| **lingual** | *0.276* | *0.076* | *-0.089* | *0.069* | 1.99 |
| **postcentral** | **0.412* | *0.014* | *-0.017* | *0.146* | 2.15 |
| **lateraloccipital** | **0.451* | *0.124* | *-0.220* | **0.498* | 2.22 |
| **superiorparietal** | **0.502* | *0.146* | *-0.329* | *0.265* | 2.23 |
| **isthmuscingulate** | *0.273* | *-0.037* | *-0.230* | *-0.133* | 2.35 |
| **precuneus** | **0.462* | *0.010* | *-0.335* | **-0.557* | 2.46 |
| **transversetemporal** | *0.278* | *0.150* | *-0.121* | *0.238* | 2.49 |
| **rostralmiddlefrontal** | **0.509* | **0.451* | *0.351* | **0.509* | 2.51 |
| **paracentral** | *0.133* | *0.095* | *-0.076* | *-0.230* | 2.51 |
| **parahippocampal** | *-0.188* | *0.007* | *0.287* | *0.037* | 2.57 |
| **posteriorcingulate** | *0.300* | *0.210* | *0.153* | *-0.229* | 2.58 |
| **inferiorparietal** | **0.401* | *0.349* | *0.089* | *0.100* | 2.59 |
| **caudalanteriorcingulate** | *0.233* | **0.387* | *0.071* | *0.056* | 2.59 |
| **parstriangularis** | *0.045* | **0.528* | **0.464* | *-0.006* | 2.61 |
| **supramarginal** | ***0.623* | *0.302* | *-0.132* | *-0.080* | 2.63 |
| **bankssts** | *0.212* | *-0.042* | *-0.039* | *-0.029* | 2.68 |
| **frontalpole** | *0.013* | *0.105* | *0.181* | *0.039* | 2.69 |
| **medialorbitofrontal** | *0.364* | *0.257* | *0.056* | *-0.002* | 2.71 |
| **precentral** | *0.302* | *0.167* | *-0.005* | *0.132* | 2.71 |
| **caudalmiddlefrontal** | **0.46* | *0.346* | *0.026* | *0.289* | 2.72 |
| **fusiform** | *0.161* | *0.013* | *-0.039* | *-0.106* | 2.78 |
| **lateralorbitofrontal** | *0.039* | *0.206* | *0.285* | *0.153* | 2.79 |
| **parsopercularis** | *0.247* | **0.424* | **0.483* | *0.154* | 2.80 |
| **superiorfrontal** | ***0.747* | **0.462* | *0.246* | ***0.618* | 2.82 |
| **parsorbitalis** | *0.287* | *0.083* | *-0.045* | *0.160* | 2.86 |
| **inferiortemporal** | *-0.184* | *-0.008* | *0.368* | **-0.422* | 2.91 |
| **superiortemporal** | *0.068* | *0.140* | *0.280* | *-0.087* | 2.93 |
| **rostralanteriorcingulate** | *0.205* | **0.486* | *0.200* | *-0.084* | 2.99 |
| **middletemporal** | *-0.041* | *0.047* | **0.440* | **-0.436* | 3.06 |
| **insula** | *-0.053* | *0.357* | **0.567* | *-0.072* | 3.11 |
| **entorhinal** | *0.063* | *-0.141* | *-0.247* | *0.067* | 3.31 |
| **temporalpole** | *0.240* | *-0.004* | *-0.074* | *0.115* | 3.74 |
| ***Mean*** | **0.27** | **0.17** | **0.07** | **0.04** | **2.63** |
| ***SD*** | **0.25** | **0.20** | **0.27** | **0.27** | **0.40** |
| ***p-value*** | **< 0.0001** | **< 0.0001** | **0.1523** | **0.4108** |  |

**Supplementary Table 10.** Columns 2–5 show correlations between mean cortical distance and regional lFCD, CMRglc, rCST, and rPWR across subjects (cohort-1, *n* = 28). Regions are sorted by their mean cortical distance (last column) (**p*< 0.05, ***p*< 0.05 Bonferroni). Mean and SD and significance levels are reported for the Fisher’s z-transformed correlations across regions.

| **Region** | **lFCD** | **CMRglc** | **rCST** | **rPWR** | **Mean Distance (mm)** |
| --- | --- | --- | --- | --- | --- |
| **posteriorcingulate** | *0.241* | *-0.371* | **-0.545* | *-0.020* | 83.75 |
| **isthmuscingulate** | *0.150* | *-0.365* | *-0.221* | *-0.235* | 85.56 |
| **caudalanteriorcingulate** | *-0.119* | *-0.345* | *-0.060* | *-0.230* | 88.46 |
| **insula** | *-0.038* | *-0.177* | *0.176* | **0.413* | 90.31 |
| **parahippocampal** | *-0.136* | *-0.179* | *0.060* | *-0.076* | 91.06 |
| **transversetemporal** | *0.212* | *-0.127* | *-0.275* | *0.291* | 93.38 |
| **paracentral** | *0.063* | *-0.349* | *-0.211* | *0.078* | 96.28 |
| **entorhinal** | **-0.558* | *0.019* | ***0.583* | *-0.222* | 97.85 |
| **precuneus** | *0.199* | *-0.241* | *-0.266* | *-0.052* | 98.04 |
| **rostralanteriorcingulate** | *-0.196* | *-0.100* | *0.130* | *0.026* | 98.83 |
| **precentral** | *0.020* | *-0.204* | *-0.100* | *0.193* | 99.33 |
| **superiorfrontal** | *-0.133* | *-0.293* | *-0.087* | *-0.293* | 101.26 |
| **fusiform** | *0.111* | *-0.048* | *0.148* | *0.269* | 101.27 |
| **superiortemporal** | *-0.069* | *-0.150* | *0.240* | **0.485* | 102.09 |
| **medialorbitofrontal** | *-0.302* | *-0.018* | *0.125* | *-0.221* | 102.52 |
| **parsopercularis** | *0.045* | *-0.146* | *0.143* | *0.244* | 102.52 |
| **postcentral** | *0.094* | *-0.084* | *-0.094* | **0.406* | 102.52 |
| **lingual** | *0.136* | *-0.202* | *0.069* | *0.117* | 103.21 |
| **lateralorbitofrontal** | **-0.506* | *0.124* | ***0.596* | **0.507* | 103.36 |
| **caudalmiddlefrontal** | *-0.366* | *-0.226* | *0.293* | *-0.358* | 104.12 |
| **temporalpole** | **-0.455* | *-0.042* | **0.461* | *0.031* | 107.54 |
| **bankssts** | *0.229* | *-0.044* | *-0.145* | *0.346* | 107.67 |
| **supramarginal** | *0.292* | *-0.114* | **-0.376* | *0.284* | 108.24 |
| **middletemporal** | *-0.328* | *-0.109* | **0.502* | *0.239* | 108.51 |
| **inferiortemporal** | *-0.357* | *-0.088* | ***0.597* | *-0.017* | 108.99 |
| **cuneus** | *0.020* | **-0.376* | *0.056* | *-0.256* | 110.56 |
| **superiorparietal** | *-0.099* | *-0.284* | *0.083* | *-0.327* | 111.56 |
| **parstriangularis** | **0.406* | *-0.164* | *-0.299* | ***0.580* | 111.63 |
| **pericalcarine** | *0.044* | *-0.317* | *0.182* | *-0.235* | 112.17 |
| **rostralmiddlefrontal** | *-0.142* | *-0.040* | *0.237* | *0.089* | 114.88 |
| **inferiorparietal** | *0.140* | *-0.080* | *-0.173* | *0.117* | 115.75 |
| **parsorbitalis** | *-0.089* | *0.018* | *0.175* | *0.236* | 118.77 |
| **lateraloccipital** | *0.156* | *0.029* | *0.070* | **0.457* | 125.14 |
| **frontalpole** | *-0.078* | *0.193* | *0.179* | *0.013* | 129.26 |
| ***Mean*** | **-0.05** | **-0.15** | **0.07** | **0.09** | **104.01** |
| ***SD*** | **0.25** | **0.15** | **0.31** | **0.29** | **10.36** |
| ***p-value*** | **0.2914** | **< 0.0001** | **0.1721** | **0.069** |  |

**Supplementary Table 11.** Columns 2–5 show correlations between fMRI tSNR and regional lFCD, CMRglc, rCST, and rPWR across subjects (cohort-1, *n* = 28). Regions are sorted by their mean fMRI tSNR (last column) (**p*< 0.05, ***p*< 0.05 Bonferroni). Mean and SD and significance levels are reported for the Fisher’s z-transformed correlations across regions.

| **Region** | **lFCD** | **CMRglc** | **rCST** | **rPWR** | **Mean fMRI tSNR** |
| --- | --- | --- | --- | --- | --- |
| **temporalpole** | *-0.143* | *0.120* | *0.201* | *0.325* | 12.91 |
| **entorhinal** | *-0.266* | *0.156* | *0.265* | *0.134* | 17.51 |
| **frontalpole** | *0.177* | *-0.185* | *-0.082* | *0.114* | 18.23 |
| **medialorbitofrontal** | *-0.004* | *-0.089* | *-0.127* | *0.299* | 20.32 |
| **lateralorbitofrontal** | **-0.547* | *0.142* | **0.438* | **0.374* | 24.07 |
| **inferiortemporal** | *-0.311* | *0.255* | **0.521* | *-0.050* | 26.11 |
| **parsorbitalis** | *-0.265* | *0.231* | **0.528* | **0.490* | 27.10 |
| **middletemporal** | *-0.210* | *0.132* | *0.323* | *0.129* | 31.88 |
| **parahippocampal** | *-0.066* | *0.170* | *0.364* | *0.118* | 31.94 |
| **fusiform** | *-0.216* | *0.006* | *0.143* | *-0.142* | 34.42 |
| **superiortemporal** | *-0.076* | *0.306* | *0.156* | *0.163* | 37.84 |
| **lateraloccipital** | *-0.141* | *-0.149* | *-0.141* | *-0.073* | 42.22 |
| **lingual** | *-0.254* | *-0.088* | *-0.023* | *-0.203* | 43.48 |
| **parstriangularis** | *0.179* | *-0.113* | *-0.183* | **0.389* | 43.51 |
| **rostralanteriorcingulate** | *-0.030* | *0.052* | *-0.157* | *0.125* | 44.73 |
| **transversetemporal** | *-0.317* | *0.326* | *0.352* | *-0.126* | 45.56 |
| **insula** | *-0.231* | **0.387* | **0.451* | *0.116* | 48.37 |
| **rostralmiddlefrontal** | *0.183* | *0.065* | *-0.339* | **0.392* | 49.10 |
| **bankssts** | *0.019* | *0.196* | *-0.178* | *-0.065* | 49.43 |
| **parsopercularis** | *0.182* | *0.144* | *-0.060* | **0.477* | 49.58 |
| **postcentral** | *-0.204* | *-0.006* | *-0.232* | *-0.357* | 49.76 |
| **precentral** | *-0.060* | *0.130* | *-0.291* | *-0.110* | 51.78 |
| **supramarginal** | *0.002* | *0.154* | **-0.455* | *-0.045* | 52.60 |
| **isthmuscingulate** | *-0.084* | *0.200* | *-0.016* | *0.203* | 54.27 |
| **pericalcarine** | **-0.449* | *0.069* | *0.159* | *-0.110* | 54.72 |
| **inferiorparietal** | *0.155* | *0.114* | *-0.366* | **0.382* | 55.81 |
| **cuneus** | **-0.459* | *-0.100* | *0.228* | **-0.410* | 56.90 |
| **caudalmiddlefrontal** | *0.307* | *0.238* | *-0.334* | **0.515* | 57.29 |
| **superiorfrontal** | *0.260* | *0.209* | *-0.288* | **0.489* | 57.51 |
| **caudalanteriorcingulate** | *0.039* | *0.232* | *0.192* | *0.276* | 59.08 |
| **posteriorcingulate** | *-0.134* | **0.401* | *0.177* | *0.062* | 59.25 |
| **paracentral** | *-0.361* | *0.034* | *0.097* | **-0.502* | 59.30 |
| **superiorparietal** | *-0.144* | *-0.213* | *-0.348* | *-0.305* | 61.26 |
| **precuneus** | *-0.271* | *0.108* | *-0.174* | *-0.079* | 63.31 |
| ***Mean*** | **-0.12** | **0.11** | **0.03** | **0.09** | **43.86** |
| ***SD*** | **0.23** | **0.16** | **0.30** | **0.29** | **14.40** |
| ***p-value*** | **0.0051** | **0.0004** | **0.602** | **0.0703** |  |

**Supplementary Table 12.** Columns 2–5 show correlations between FDG-PET tSNR and regional lFCD, CMRglc, rCST, and rPWR across subjects (cohort-1, *n* = 28). Regions are sorted by their mean FDG-PET tSNR (last column) (**p*< 0.05, ***p*< 0.05 Bonferroni). Mean and SD and significance levels are reported for the Fisher’s z-transformed correlations across regions.

| **Region** | **lFCD** | **CMRglc** | **rCST** | **rPWR** | **Mean FDG-PET tSNR** |
| --- | --- | --- | --- | --- | --- |
| **entorhinal** | *0.066* | **0.494* | *0.026* | *0.124* | 2.31 |
| **temporalpole** | *0.245* | **0.495* | *-0.069* | *0.338* | 2.37 |
| **parahippocampal** | *0.051* | **0.554* | *-0.026* | *0.046* | 2.50 |
| **fusiform** | *-0.055* | **0.504* | *-0.130* | *-0.193* | 2.70 |
| **inferiortemporal** | *-0.056* | ***0.611* | *0.133* | *0.172* | 2.77 |
| **paracentral** | *-0.239* | *0.237* | **0.387* | *-0.109* | 2.78 |
| **insula** | *-0.152* | **0.546* | *0.302* | *-0.105* | 2.82 |
| **caudalanteriorcingulate** | *-0.125* | **0.554* | **0.517* | *0.326* | 2.84 |
| **superiortemporal** | *-0.036* | **0.492* | *0.015* | *-0.093* | 2.88 |
| **isthmuscingulate** | *-0.111* | ***0.600* | *0.343* | **0.488* | 2.88 |
| **lingual** | *-0.054* | **0.437* | *-0.185* | *-0.050* | 2.89 |
| **postcentral** | *-0.252* | *0.117* | *0.133* | **-0.396* | 2.89 |
| **rostralanteriorcingulate** | *-0.088* | ***0.574* | *0.203* | *0.220* | 2.92 |
| **middletemporal** | *-0.037* | **0.561* | *0.052* | *0.097* | 2.93 |
| **precentral** | *-0.197* | *0.257* | *0.207* | *-0.238* | 2.93 |
| **posteriorcingulate** | *-0.258* | **0.394* | **0.501* | *0.061* | 2.94 |
| **medialorbitofrontal** | *0.201* | ***0.641* | *0.027* | **0.441* | 2.95 |
| **lateralorbitofrontal** | *-0.073* | ***0.580* | *0.153* | *0.234* | 2.95 |
| **superiorfrontal** | *0.138* | **0.424* | *0.190* | *0.372* | 2.99 |
| **superiorparietal** | *-0.072* | *0.171* | *-0.096* | *0.031* | 3.00 |
| **supramarginal** | *-0.063* | *0.295* | *-0.164* | *-0.053* | 3.04 |
| **caudalmiddlefrontal** | *0.308* | **0.405* | *-0.145* | **0.462* | 3.05 |
| **precuneus** | *-0.275* | *0.319* | *0.239* | *-0.126* | 3.06 |
| **bankssts** | *0.078* | **0.440* | *-0.337* | *-0.065* | 3.08 |
| **pericalcarine** | *-0.049* | **0.488* | *-0.076* | *0.215* | 3.09 |
| **parstriangularis** | *-0.341* | **0.554* | **0.528* | *-0.106* | 3.12 |
| **lateraloccipital** | *0.033* | *0.330* | **-0.494* | *-0.100* | 3.12 |
| **parsorbitalis** | *-0.211* | **0.566* | **0.422* | *0.191* | 3.14 |
| **parsopercularis** | *0.009* | **0.547* | *0.243* | *0.220* | 3.16 |
| **transversetemporal** | *-0.140* | **0.523* | *0.291* | *0.010* | 3.16 |
| **inferiorparietal** | *-0.118* | **0.414* | *0.067* | *0.106* | 3.17 |
| **frontalpole** | *-0.070* | **0.492* | *0.085* | *0.049* | 3.18 |
| **cuneus** | *-0.040* | **0.398* | *-0.128* | *0.092* | 3.21 |
| **rostralmiddlefrontal** | *-0.045* | **0.515* | *0.357* | *0.156* | 3.26 |
| ***Mean*** | **-0.06** | **0.50** | **0.11** | **0.09** | **2.94** |
| ***SD*** | **0.15** | **0.16** | **0.26** | **0.22** | **0.22** |
| ***p-value*** | **0.0239** | **< 0.0001** | **0.0181** | **0.0269** |  |

**Supplementary Table 13.** Main effect of Alcohol on rCST (cluster-size corrected, *p*_FWE_ < 0.01, cluster defining threshold: *p* < 0.005, *k* > 120). Coordinates are in MNI space (voxel size = 3-mm isotropic).

| **Region(s)** | **L/R** | **Contrast** | **Brodmann area(s)** | **Cluster size** | **Peak coordinates (*x*, *y*, *z*)_mm_** | | | **Peak *t*-value** | |
| --- | --- | --- | --- | --- | --- | --- | --- | --- | --- |
| **Calcarine**  **Cuneus**  **Lingual Gyrus** | L/R  L/R  L/R | ALC < PLC | 17, 18, 30 | 466 | -6 | -84 | 0 | | 5.82 |

**Supplementary Table 14.** Main effect of Alcohol on rPWR (*p*_FWE_ < 0.01, cluster-size corrected, cluster defining threshold: *p* < 0.005, *k* > 120). Coordinates are in MNI space (voxel size = 3-mm isotropic).

| **Region(s)** | **L/R** | **Contrast** | **Brodmann area(s)** | **Cluster size** | **Peak coordinates (*x*, *y*, *z*)_mm_** | | | **Peak *t*-value** | |
| --- | --- | --- | --- | --- | --- | --- | --- | --- | --- |
| **Calcarine**  **Cuneus**  **Lingual Gyrus** | L/R  L/R  L/R | ALC < PLC | 17, 18 | 466 | 0 | -84 | -6 | | 5.69 |
| **Thalamus**  ***Ventral Lateral Nucleus***  ***Medial Dorsal Nucleus*** | L/R | PLC < ALC | - | 185 | -12 | -24 | 0 | | 4.98 |

**Supplementary Table 15.** Main effect of Group on rCST (cluster-size corrected, *p*_FWE_ < 0.01, cluster defining threshold: *p* < 0.005, *k* > 120). Coordinates are in MNI space (voxel size = 3-mm isotropic).

| **Region(s)** | **L/R** | **Contrast** | **Brodmann area(s)** | **Cluster size** | **Peak coordinates (*x*, *y*, *z*)_mm_** | | | **Peak *t*-value** | |
| --- | --- | --- | --- | --- | --- | --- | --- | --- | --- |
| **Precuneus**  **Cuneus** | L/R  L/R | HD < LD | 7, 31 | 270 | -15 | -57 | 24 | | 7.14 |
| **Cingulate Gyrus**  **Medial Frontal Gyrus**  **Superior Frontal Gyrus** | L/R  L/R  L/R | HD < LD | 6, 24 | 317 | 0 | -18 | 45 | | 6.53 |
| **Inferior Frontal Gyrus Insula**  **Putamen** | L/R  L/R  L/R | HD < LD | 13, 45 | 190 | -33 | 12 | 9 | | 6.42 |
| **Cerebellum (Posterior Lobe)**  ***Crus I***  ***Crus II***  ***Declive*** | L | HD < LD | - | 165 | -6 | -84 | -33 | | 5.69 |
| **Cerebellum (Posterior Lobe)**  ***Crus I***  ***Declive***  ***Tuber*** | R | HD < LD | - | 224 | 36 | -60 | -30 | | 5.00 |
| **Calcarine**  **Posterior Cingulate**  **Middle Temporal Gyrus**  **Middle Occipital Gyrus** | L/R  L/R  L  L/R | LD < HD | 18, 19, 30 | 554 | -36 | -90 | -18 | | 6.62 |

**Supplementary Table 16.** Main effect of Group on rPWR (cluster-size corrected, *p*_FWE_ < 0.01, cluster defining threshold: *p* < 0.005, *k* > 120). Coordinates are in MNI space (voxel size = 3-mm isotropic).

| **Region(s)** | **L/R** | **Contrast** | **Brodmann area(s)** | **Cluster size** | **Peak coordinates (*x*, *y*, *z*)_mm_** | | | **Peak *t*-value** | |
| --- | --- | --- | --- | --- | --- | --- | --- | --- | --- |
| **Midbrain**  ***Red Nucleus***  **Pons**  **Thalamus**  ***Medial Dorsal Nucleus***  ***Pulvinar*** | L/R  L/R  L/R | HD < LD | - | 224 | 36 | -60 | -30 | | 7.83 |
| **Medial Frontal Gyrus**  **Precuneus**  **Superior Frontal Gyrus** | L/R  L/R  L/R | HD < LD | 6, 7, 8, 32 | 420 | 0 | 36 | 39 | | 7.25 |
| **Medial Frontal Gyrus**  **Superior Frontal Gyrus** | L/R  L/R | HD < LD | 10 | 317 | -3 | 66 | 9 | | 6.70 |
| **Middle Temporal Gyrus**  **Middle Occipital Gyrus** | L  L | HD < LD | 18, 19 | 222 | -30 | -96 | -15 | | 6.34 |
| **Cerebellum (Posterior Lobe)**  ***Crus I***  ***Declive***  ***Uvula*** | L/R | LD < HD | - | 569 | 0 | -87 | -36 | | 5.95 |
| **Precuneus**  **Cuneus** | L/R  L/R | LD < HD | 7, 31 | 135 | -9 | -72 | 24 | | 5.27 |

**Supplementary Table 17.** Association between rPWR and subjective experience of alcohol (subjective-PC) in light and heavy drinkers (*n* = 38) in cohort-2 (*p*_FWE_ < 0.01, cluster-size corrected, cluster defining threshold: *p* < 0.005). Coordinates are in MNI space (voxel size = 3-mm isotropic).

| **Region(s)** | **L/R** | **Contrast** | **Brodmann area(s)** | **Cluster size** | **Peak coordinates (*x*, *y*, *z*)_mm_** | | | **Peak *t*-value** | |
| --- | --- | --- | --- | --- | --- | --- | --- | --- | --- |
| **Middle Temporal Gyrus**  **Inferior Temporal Gyrus** | R  R | Subjective-PC | 20, 21 | 60 | 60 | -45 | -18 | | -4.60 |
| **Insula**  **Putamen**  **Claustrum** | R  R  R | Subjective-PC | 13 | 61 | 36 | -3 | -3 | | -5.18 |

**Supplementary Table 18.** Association between rCST and the cognitive-PC in heavy drinkers (*n* = 15) in cohort-2 (*p*_FWE_ < 0.01, cluster-size corrected, cluster defining threshold: *p* < 0.005). Coordinates are in MNI space (voxel size = 3-mm isotropic). At lower uncorrected thresholds this effect was bilateral.

| **Region(s)** | **L/R** | **Contrast** | **Brodmann area(s)** | **Cluster size** | **Peak coordinates (x, y, z)_mm_** | | | **Peak *t*-value** | |
| --- | --- | --- | --- | --- | --- | --- | --- | --- | --- |
| **Inferior Parietal Lobule**  **Supramarginal Gyrus** | R | Cognitive-PC | 40 | 77 | 57 | -42 | 45 | | 8.63 |

**Supplementary References**

1. Cha Y-HK, Jog MA, Kim Y-C, Chakrapani S, Kraman SM, Wang DJ. Regional correlation between resting state FDG PET and pCASL perfusion MRI. *Journal of Cerebral Blood Flow & Metabolism* **33**, 1909-1914 (2013).

2. Glasser MF*, et al.* The minimal preprocessing pipelines for the Human Connectome Project. *NeuroImage* **80**, 105-124 (2013).

3. Desikan RS*, et al.* An automated labeling system for subdividing the human cerebral cortex on MRI scans into gyral based regions of interest. *Neuroimage* **31**, 968-980 (2006).

4. Fischl B, Dale AM. Measuring the thickness of the human cerebral cortex from magnetic resonance images. *Proceedings of the National Academy of Sciences* **97**, 11050-11055 (2000).

5. Phelps M, Huang S, Hoffman E, Selin C, Sokoloff L, Kuhl D. Tomographic measurement of local cerebral glucose metabolic rate in humans with (F‐18) 2‐fluoro‐2‐deoxy‐D‐glucose: validation of method. *Annals of neurology* **6**, 371-388 (1979).

6. Huang S-C, Phelps ME, Hoffman EJ, Sideris K, Selin CJ, Kuhl DE. Noninvasive determination of local cerebral metabolic rate of glucose in man. *American Journal of Physiology-Endocrinology And Metabolism* **238**, E69-E82 (1980).

7. Friston KJ*, et al.* Analysis of fMRI time-series revisited. *NeuroImage* **2**, 45-53 (1995).

8. Shokri-Kojori E, Tomasi D, Wiers CE, Wang GJ, Volkow ND. Alcohol affects brain functional connectivity and its coupling with behavior: greater effects in male heavy drinkers. *Molecular Psychiatry* **22**, 1185 (2016).

9. Sokoloff L*, et al.* The [14C] deoxyglucose method for the measurement of local cerebral glucose utilization: theory, procedure, and normal values in the conscious and anesthetized albino rat. *Journal of neurochemistry* **28**, 897-916 (1977).

10. Volkow ND, Fowler JS, Wolf AP. Changes in brain glucose metabolism in cocaine dependence and withdrawal. *American Journal of Psychiatry* **148**, 621-626 (1991).

11. Power JD, Barnes KA, Snyder AZ, Schlaggar BL, Petersen SE. Spurious but systematic correlations in functional connectivity MRI networks arise from subject motion. *NeuroImage* **59**, 2142-2154 (2012).

12. Tomasi D, Volkow ND. Functional connectivity density mapping. *Proc Natl Acad Sci U S A* **107**, 9885-9890 (2010).

13. Zang Y, Jiang T, Lu Y, He Y, Tian L. Regional homogeneity approach to fMRI data analysis. *Neuroimage* **22**, 394-400 (2004).

14. Zou Q-H*, et al.* An improved approach to detection of amplitude of low-frequency fluctuation (ALFF) for resting-state fMRI: fractional ALFF. *Journal of neuroscience methods* **172**, 137-141 (2008).

15. Biswal BB, Kannurpatti SS, Rypma B. Hemodynamic scaling of fMRI-BOLD signal: validation of low-frequency spectral amplitude as a scalability factor. *Magnetic resonance imaging* **25**, 1358-1369 (2007).

16. Tomasi D, Shokri-Kojori E, Volkow N. Temporal changes in local functional connectivity density reflect the temporal variability of the amplitude of low frequency fluctuations in gray matter. *PLoS One* **11**, e0154407 (2016).

17. Zhang XD*, et al.* Decreased coupling between functional connectivity density and amplitude of low frequency fluctuation in non-neuropsychiatric systemic lupus erythematosus: a resting-stage functional MRI study. *Molecular neurobiology* **54**, 5225-5235 (2017).

18. Thompson GJ, Riedl V, Grimmer T, Drzezga A, Herman P, Hyder F. The whole-brain “global” signal from resting state fMRI as a potential biomarker of quantitative state changes in glucose metabolism. *Brain connectivity* **6**, 435-447 (2016).

19. Grinvald A, Lieke EE, Frostig RD, Hildesheim R. Cortical point-spread function and long-range lateral interactions revealed by real-time optical imaging of macaque monkey primary visual cortex. *Journal of Neuroscience* **14**, 2545-2568 (1994).

20. Rosenbaum R, Smith MA, Kohn A, Rubin JE, Doiron B. The spatial structure of correlated neuronal variability. *Nature Neuroscience*, (2016).

21. Caliński T, Harabasz J. A dendrite method for cluster analysis. *Communications in Statistics-theory and Methods* **3**, 1-27 (1974).

22. Huisman MC*, et al.* Cerebral blood flow and glucose metabolism in healthy volunteers measured using a high-resolution PET scanner. *EJNMMI research* **2**, 63 (2012).
